# Bidirectional Relationship Between Reduced Blood pH and Acute Pancreatitis: A Translational Study of Their Noxious Combination

**DOI:** 10.3389/fphys.2018.01360

**Published:** 2018-10-01

**Authors:** Zoltan Rumbus, Emese Toth, Laszlo Poto, Aron Vincze, Gabor Veres, Laszlo Czako, Emoke Olah, Katalin Marta, Alexandra Miko, Zoltan Rakonczay, Zsolt Balla, Jozsef Kaszaki, Imre Foldesi, Jozsef Maleth, Peter Hegyi, Andras Garami

**Affiliations:** ^1^Institute for Translational Medicine, Medical School, University of Pecs, Pecs, Hungary; ^2^Momentum Gastroenterology Multidisciplinary Research Group, Hungarian Academy of Sciences–University of Szeged, Szeged, Hungary; ^3^First Department of Medicine, University of Szeged, Szeged, Hungary; ^4^Institute of Bioanalysis, Medical School, University of Pecs, Pecs, Hungary; ^5^Department of Gastroenterology, First Department of Medicine, University of Pecs, Pecs, Hungary; ^6^First Department of Pediatrics, Semmelweis University, Budapest, Hungary; ^7^Department of Translational Medicine, First Department of Medicine, University of Pecs, Pecs, Hungary; ^8^Department of Pathophysiology, University of Szeged, Szeged, Hungary; ^9^Institute of Surgical Research, University of Szeged, Szeged, Hungary; ^10^Department of Laboratory Medicine, University of Szeged, Szeged, Hungary; ^11^Momentum Epithel Cell Signaling and Secretion Research Group, Hungarian Academy of Sciences–University of Szeged, Szeged, Hungary

**Keywords:** experimental pancreatitis, acidosis, acid-base balance, meta-analysis, mortality

## Abstract

Acute pancreatitis (AP) is often accompanied by alterations in the acid-base balance, but how blood pH influences the outcome of AP is largely unknown. We studied the association between blood pH and the outcome of AP with meta-analysis of clinical trials, and aimed to discover the causative relationship between blood pH and AP in animal models. PubMed, EMBASE, and Cochrane Controlled Trials Registry databases were searched from inception to January 2017. Human studies reporting systemic pH status and outcomes (mortality rate, severity scores, and length of hospital stay) of patient groups with AP were included in the analyses. We developed a new mouse model of chronic metabolic acidosis (MA) and induced mild or severe AP in the mice. Besides laboratory blood testing, the extent of pancreatic edema, necrosis, and leukocyte infiltration were assessed in tissue sections of the mice. Thirteen studies reported sufficient data in patient groups with AP (*n* = 2,311). Meta-analysis revealed markedly higher mortality, elevated severity scores, and longer hospital stay in AP patients with lower blood pH or base excess (*P* < 0.001 for all studied outcomes). Meta-regression analysis showed significant negative correlation between blood pH and mortality in severe AP. In our mouse model, pre-existing MA deteriorated the pancreatic damage in mild and severe AP and, vice versa, severe AP further decreased the blood pH of mice with MA. In conclusion, MA worsens the outcome of AP, while severe AP augments the decrease of blood pH. The discovery of this vicious metabolic cycle opens up new therapeutic possibilities in AP.

## Introduction

Acute pancreatitis (AP) is one of the most frequent gastrointestinal causes of hospitalization with significant morbidity and mortality in the US (Yadav and Lowenfels, 2013; Parniczky et al., 2016). Although the mortality rate in mild and moderate AP is low, this value is still unacceptably high (30%) in its severe form (Parniczky et al., 2016). Since no specific therapy is available, only prompt and accurate interventions, such as aggressive fluid therapy can be beneficial (Vinish et al., 2017).

An important function of the pancreas is bicarbonate production, which is required to maintain its constant “milieu intérieur,” thereby to prevent premature activation of pancreatic proteases (Pallagi et al., 2011, 2015; Hegyi and Petersen, 2013). When pancreatic bicarbonate production is challenged by local or systemic acid load (i.e., metabolic acidosis, MA), the resulting lower pH can facilitate pancreatic enzyme activation and deteriorate cell damage (Reed et al., 2011). Furthermore, injection of acidic contrast solution either into the pancreatic duct or into the vein significantly increased the severity of AP in rats (Noble et al., 2008; Bhoomagoud et al., 2009). Beside an external acid load, the pancreatic pH balance can also be compromised by tissue injury such as AP, which can lead to acidification of local tissues, thus deteriorate cell damage (Behrendorff et al., 2010). The luminal pH of the main pancreatic duct was also lower in human patients with AP compared to controls (Takacs et al., 2013), suggesting that the development of AP is accompanied by a reduction of local pH. Multiple mechanisms have been implicated in AP which can lead to MA, including direct mechanisms such as the loss of bicarbonate-rich pancreatic juice via pancreatic fistula or drainage (Rice et al., 2014), as well as indirect ones through lactic acidosis which can sequentially occur in AP due to shock, sepsis, cardiovascular failure, or upper gastrointestinal bleeding (Zhan et al., 2015). However, the interaction between AP and systemic pH is still not fully clarified.

Acidosis is often considered as a marker of disease severity, viz., a by-product of systemic dysregulation, and as such it is a proven prognostic factor in the assessment of critically ill patients (Vincent and Moreno, 2010). Despite the fact that scoring systems, which are used to help the diagnosis and the assessment of the progression of AP, include the changes in systemic pH balance of the patients (e.g., Acute Physiology and Chronic Health Evaluation, APACHE II and Ranson scores), clinical trials aiming to reveal a correlation between the acid-base status and the outcome of AP are scarce. To our knowledge, the sole published human study, which aimed to directly answer this question showed that changes in the parameters of systemic acid-base status can predict mortality in AP (Sharma et al., 2014). On the contrary, the necessity of arterial blood gas sampling was questioned in patients with AP in another human study (Ward et al., 2008). With regards to the results obtained in experimental animals, a detailed analysis of the correlation between systemic pH and the outcome of AP would be of utmost importance, because it could establish blood pH as a predictor of the severity and the outcome of the disease and, arguably, identify acidosis as a therapeutic target in AP.

In the present study, by using a dual, translational approach, we have discovered a vicious, bidirectional interaction between blood pH and the outcome of AP. Based on our discovery, the possibility of new therapeutic approaches in AP can be suggested.

## Materials and methods

### Study design 1: meta-analysis of clinical trials

#### Search strategy

Our meta-analysis was conducted in accordance with the guidelines of the Preferred Reporting Items for Systematic Reviews and Meta-Analysis Protocols (Moher et al., 2009) (Supplementary Table 1), similarly as in our recent study (Olah et al., 2018). The analysis was based on the Patients, Intervention (or indicator), Comparison, Outcome (PICO) model: in patients with AP, we aimed to assess the predictive role of the change in pH status (as assessed by blood pH, bicarbonate concentration, base excess, or base deficit) on disease severity (indicated by clinical scores), length of hospital stay (LOS), and mortality ratio. This meta-analysis has been registered with PROSPERO (CRD42017055396).

A search in the PubMed, EMBASE, and Cochrane Controlled Trials Registry databases was performed from inception to January 2017 using the following terms: “pancreatitis AND (mortality OR survival OR severity) AND (“arterial pH” OR “blood pH” OR “systemic pH” OR “base deficit” OR “base excess” OR bicarbonate OR HCO3- OR “anion gap” OR acidosis OR alkalosis OR acid-base).” We restricted our search to original human studies published in English without time period limitations. A manual search of the reference lists of relevant full-text articles was conducted to identify further potentially eligible articles. The search was conducted separately by two authors (ZRu, AG), who also assessed study eligibility and extracted data from the selected studies independently. Disagreements were resolved by consensus with the help of a third party (PH).

#### Study selection and data extraction

The titles and abstracts of the publications from the literature search were screened and the full text of potentially eligible articles was obtained. We included studies in which blood pH or a related parameter (e.g., base excess, base deficit, or bicarbonate) and severity scores or LOS or mortality ratios were reported for the same group(s) of patients with AP. From all included articles we extracted the sample size, the reported mean pH or its related parameter for the studied patient groups with the corresponding standard error (SEM) or deviation, as well as the severity score, LOS, and mortality ratio within the group. To analyze the influence of the change in acid-base status on the severity and the outcome of AP, in each study we assigned the patient groups as a lower pH group and as a higher pH group, irrespective from the original basis for grouping used by the authors of the study.

#### Outcomes of interest

We used mortality ratio of the AP patients groups as the primary outcome. Regarding secondary outcomes, we used two commonly applied severity indices (i.e., APACHE II and Ranson scores) and the LOS.

#### Quality assessment

We assessed the quality of each study included in the meta-analysis by using the Newcastle-Ottawa Scale (Wells et al., 2000; Supplementary Table 2).

#### Statistical analysis

We used logit transformation of event rates for mortality ratios and standardized mean difference (SMD) for LOS and severity scores as the effect size data. The secondary outcomes were compared between the lower and higher pH groups (see above) within each study, and then the estimated pooled mean values were calculated. The relevant studies were compared with standard meta-analysis tools (e.g., forest plot) in case of each outcome.

Between-study heterogeneity was assessed by I^2^ statistical test, where I^2^ is the proportion of total variation attributable to between-study variability (an I^2^ value of more than 50 was considered as indication of considerable statistical heterogeneity). The selection of patients, study design, and the used methods showed variability among the studies included in our analyses, which also resulted in statistical heterogeneity. Since the lack of statistical significant results on these heterogeneity tests could be also due to the lack of power because of the small number of studies eligible for the analyses, we used the random effect model in case of each forest plot, similarly to our earlier meta-analysis (Rumbus et al., 2017). Publication bias was assessed by funnel-plot analysis, Egger's test and Duval and Tweedie trim and fill method (Supplementary Figures 1–5). Publication bias plots were used to assess whether studies with small sizes could have been missed in our analyses, however, due to the design of these tests they do not allow to firmly rule out the possibility that some papers missed the inclusion criteria of our search.

As a different statistical approach to reveal a correlation between systemic pH and mortality in moderate and severe forms of AP, we performed meta-regression analysis of those studies in which both blood pH and mortality rate were reported within the same patient group. The meta-analyses were performed with Comprehensive Meta-Analysis (version 3.3; Biostat, Inc., Engelwood, MJ, USA) and Stata (version 11.1; StataCorp, College Station, TX, USA) software.

### Study design 2: experimental procedures

#### Animals

The experiments were performed in 40 female FVB/N mice (Charles Rivers Laboratories, Wilmington, MA, USA). This commercially available, multipurpose mouse strain is characterized by excellent reproductive performance and it was repeatedly used by our group to study the mechanisms of AP (Kui et al., 2015; Maleth et al., 2015). The mice were housed in standard plastic cages kept in a room with an ambient temperature of 24°C on a 12-h light-dark cycle in the animal facility of the First Department of Medicine at the University of Szeged. The mice were allowed free access to water and standard laboratory chow for rodents (Biofarm, Zagyvaszanto, Hungary).

#### Ethics

All experiments were approved by the Institutional Animal Care and Use Committee of the University of Szeged and also by an independent committee assembled by national authorities (XII/3773/2012.). All experiments were conducted in compliance with the European Union Directive (2010/63/EU) and the Hungarian government decree (40/2013, II.14.).

#### Experimental modeling of chronic MA in mice

To develop a mouse model of chronic MA, the mice were randomly divided into the following 4 groups for a 12-day treatment: (i) ammonium chloride (NH_4_Cl) administration with drinking water (8.2 ± 0.5 ml/day/mouse) as reported in earlier studies (Galicek et al., 1981; Nowik et al., 2010); (ii) intraperitoneal (i.p.) injections of NH_4_Cl (0.5 ml, 0.28 M) on days 1 and 6; (iii) administration of NH_4_Cl with drinking water (as in group 1) and i.p. injections (as in group 2); and (iv) controls, receiving NH_4_Cl-free tap water and 2 i.p. injections of saline on days 1 and 6.

#### Experimental modeling of AP

Two different types of AP were used in this study. Mild acute pancreatitis (MAP) was induced by alcohol and fatty acid as described earlier (Huang et al., 2014; Maleth et al., 2015). Severe acute pancreatitis (SAP) was induced by the injections of cerulein (50 μg/kg, i.p.) at start time, and then at every hour for 9 h (Mareninova et al., 2006). In the chronic MA model, MAP and SAP were induced on day 12 of the acidifying treatment.

#### Laboratory measurements

Animals were euthanized by i.p. injection of sodium pentobarbital (50 mg/kg). Blood samples were collected by cardiac puncture. Serum amylase activity was measured by using a colorimetric kinetic method (Diagnosticum, Budapest, Hungary). Serum concentrations of creatinine and glucose as well as urea concentration in urine were measured with commercially available laboratory kits (Institute of Laboratory Medicine, University of Szeged). Arterial blood samples were collected in sealed plastic capillaries (170 μl), which were previously treated with lithium and heparin. Analysis of the arterial blood samples was performed by a blood gas analyzer (Cobas b221 system; Roche Ltd., Basel, Switzerland) within 1 min after blood collection at room temperature (22°C).

#### Histology

Histological evaluations were performed as described earlier (Kui et al., 2015). In brief, the extent of pancreatic edema (0: none; 1: patchy interlobular; 2: diffuse interlobular; 3: diffuse interlobular and intraacinar), necrosis (%), and leukocyte infiltration (0: none; 1: rare patchy interlobular; 2: patchy interlobular; 3: diffuse interlobular; 4: diffuse interlobular and intraacinar) were assessed in pancreatic tissue sections stained with haematoxylin and eosin under a light microscope (Zeiss Axio scope A1 microscope) at 40x magnification by an investigator who was expert in pancreas histology, however blinded to the animal's treatment group. The percentage of acinar cell necrosis was evaluated by ImageJ software (NIH, Bethesda, MD, USA).

#### Statistical analysis

Data were compared by one-way ANOVA followed by Holm-Sidak test, two-way ANOVA followed by Fischer Least Significant Difference test, or two-tailed Student's *t* test, as appropriate. SPSS 23.0 (IBM, Armonk, NY, USA) and Microsoft Excel (Microsoft Corporation, Redmond, WA, USA) software was used for statistical analysis. The effects were considered significant when *P* < 0.05. In the experimental part of the study, data are reported in the Mean ± SEM format.

## Results

### Meta-analysis

#### Study selection

**Figure 1:**
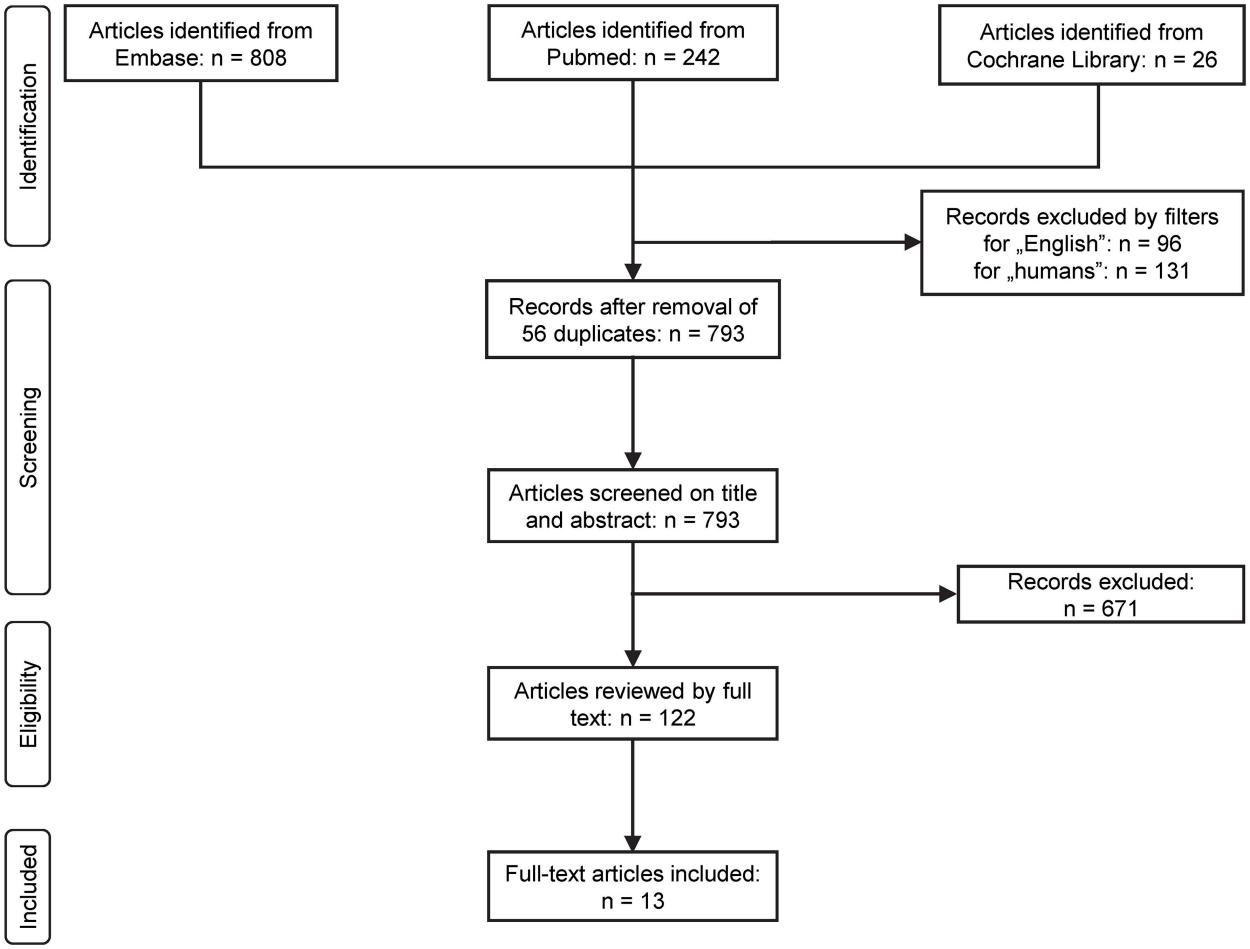
Flow chart of study selection and inclusion.

The flow chart of the study selection is presented in Figure 1. Until January 2017 the electronic literature search identified altogether 1,076 studies from the PubMed, EMBASE, and Cochrane databases. After enabling filters for human studies and English language and removal of duplicates, 793 articles remained, which were screened on title and abstract for inclusion criteria. Full texts of the remaining 122 articles were reviewed in detail. In 109 studies pH parameters or outcomes were not suitably reported in the patients with AP, therefore these were also excluded. As a result, 13 full-text publications were found eligible for statistical analysis which included data from a total of 2,311 patients (Ranson et al., 1976; Nair et al., 2000; Eachempati et al., 2002; Zhu et al., 2003; Kaya et al., 2007; Keskinen et al., 2007; Pupelis et al., 2007; De Campos et al., 2008; Shinzeki et al., 2008; Lei et al., 2013; Sharma et al., 2014; Zhan et al., 2015; Shen et al., 2016). The characteristics of these studies are summarized in Supplementary Table 3.

#### Reduction of blood pH is associated with higher mortality rate in AP

**Figure 2:**
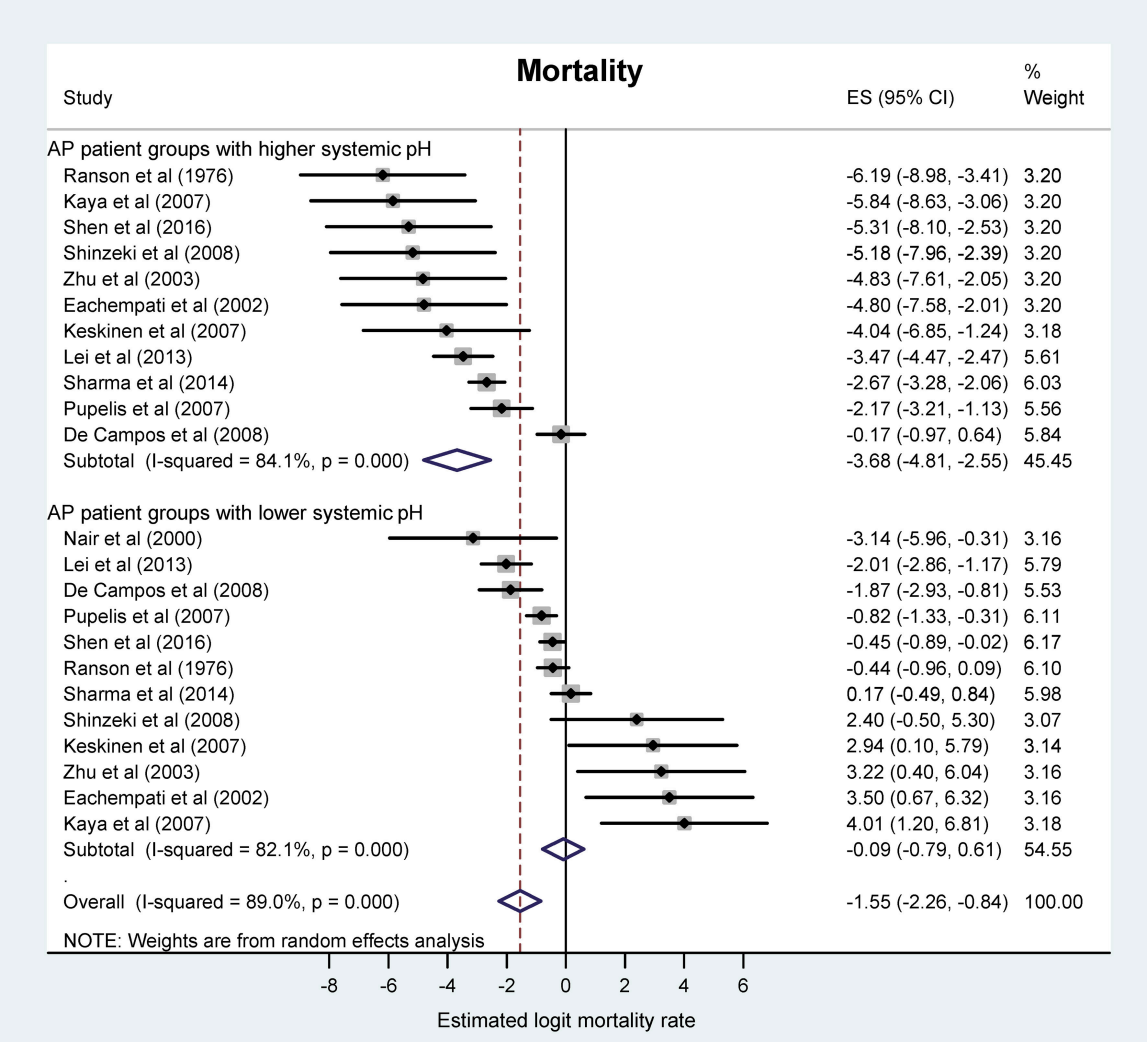
Forest plot of mortality rate using random-effects model in different systemic pH groups of patients with acute pancreatitis (AP). For each patient group, black circles and horizontal lines represent the estimated logit mortality rate (ES) and the corresponding confidence interval (CI), respectively. Lower ES corresponds with lower mortality rate and vice versa. Gray squares indicate the relative statistical weight of a given patient group. Open diamonds show the average ES and CI of patient groups with higher systemic pH (top), lower systemic pH (middle), and all patient groups (bottom).

First, we investigated the association between systemic (blood) pH status and our strongest endpoint, viz., the mortality. Our meta-analysis revealed a logit event rate of −0.09 (95% CI, −0.79, 0.61), corresponding to an average mortality rate of 51.0% (95% CI, 31.5, 70.1) in the more acidotic patient groups, while in the patient groups with higher pH or bicarbonate level the logit event rate was −3.68 (95% CI, −4.81, −2.55), which corresponds to an average mortality rate of 3.0% (95% CI, 1.2, 7.1) (Figure 2). The mortality ratios were significantly different between the two groups (*P* < 0.001).

#### Lower pH or bicarbonate concentration worsens the severity of AP

**Figure 3:**
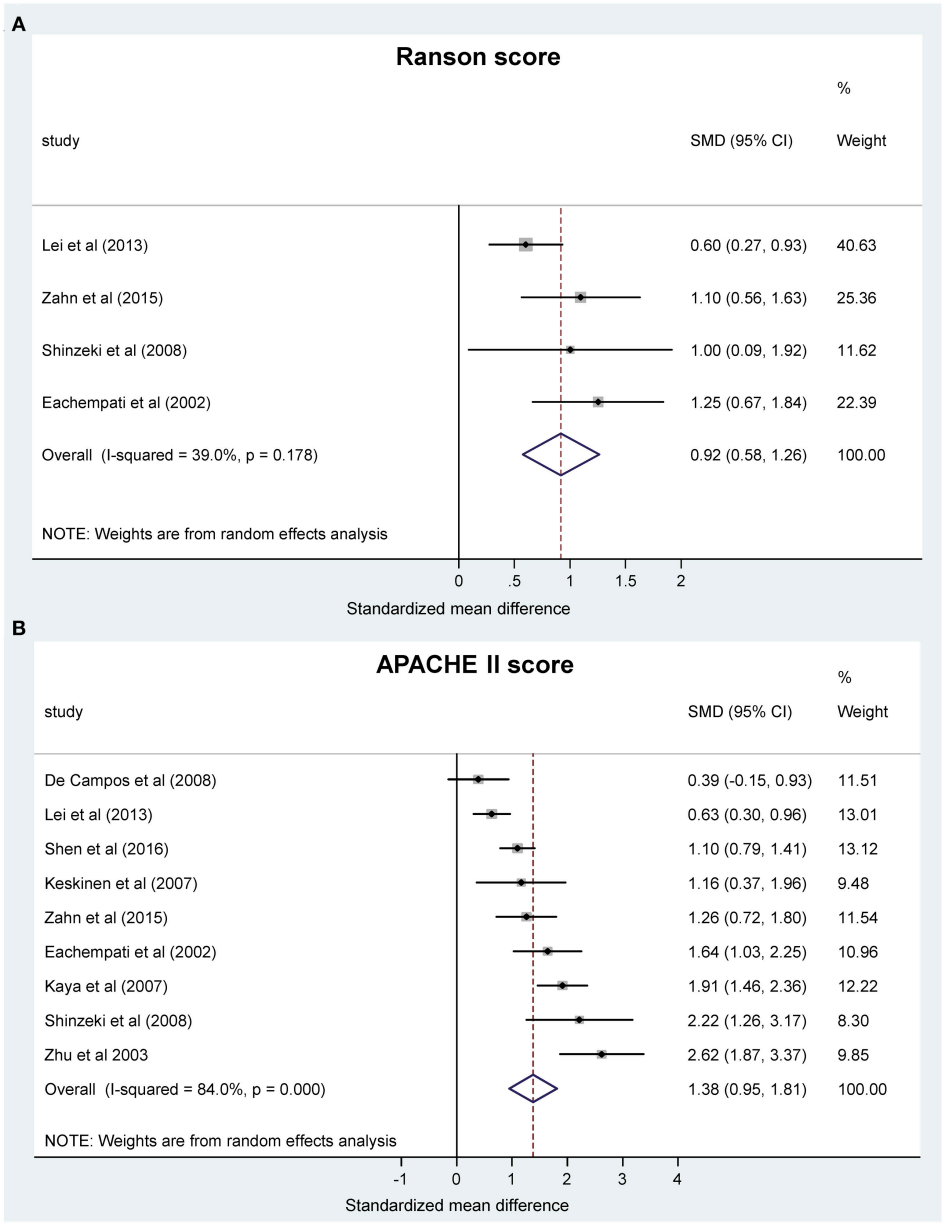
Forest plot of **(A)** Ranson scores and **(B)** Acute Physiology and Chronic Health Evaluation (APACHE II) scores using random-effects model in different systemic pH groups of patients with acute pancreatitis. Here and in Figure 4, in each study the standardized mean difference (SMD) of the outcome was calculated between the patient group with lower and higher pH. Black circles represent the SMD for each study, while the left and right horizontal arms of the circles indicate the corresponding 95% confidence intervals (CI) for the SMD for each study. The size of the gray box is proportional to the sample size of the study; bigger box represents larger sample size, thus bigger relative weight of the study, and vice versa. Circles close to zero represent smaller SMD between the lower and higher pH groups in the given study. A positive SMD means higher score (Figure 3) or longer hospital stay (Figure 4) in the patient group with lower pH compared to the patient group with higher pH. The diamond on the bottom represents the averaged SMD calculated from the SMDs of all the individual studies. The vertical dashed line is determined by the two vertical points of the diamond and indicates the value of the averaged SMD of all studies. The horizontal points of the diamond represent the 95% CI of the averaged SMD.

We wanted to know whether the change in acid-base status can also predict the severity of AP as assessed by clinical scores. Thus, we studied the association between blood pH and clinical severity scores. We found two scores, the Ranson and the APACHE II scores, which were reported in sufficient number of studies for statistical analysis (Ranson et al., 1976; Nair et al., 2000; Eachempati et al., 2002; Zhu et al., 2003; Kaya et al., 2007; Keskinen et al., 2007; Pupelis et al., 2007; De Campos et al., 2008; Shinzeki et al., 2008; Lei et al., 2013; Sharma et al., 2014; Zhan et al., 2015; Shen et al., 2016). Meta-analysis revealed that the pooled SMDs of the Ranson score (0.92, 95% CI, 0.58, 1.26) and the APACHE II score (1.38, 95% CI, 0.95, 1.81) were significantly positive between the patient groups with lower pH or bicarbonate levels and the less acidotic groups of patients (*P* < 0.001) (Figures 3A,B). These standardized values correspond to 1.60 (95% CI, 0.77, 2.42) higher Ranson score and 7.40 (95% CI, 5.05, 9.75) higher APACHE II score in the more acidotic patients with AP. The correlation found between lower blood pH and higher clinical scores could be expected as these scores also include blood pH in their calculation (Vincent and Moreno, 2010), nevertheless, these results confirm the feasibility of our meta-analysis approach to reveal an interaction between systemic pH and the outcome of AP.

#### Acidosis is associated with longer hospitalization in AP

**Figure 4:**
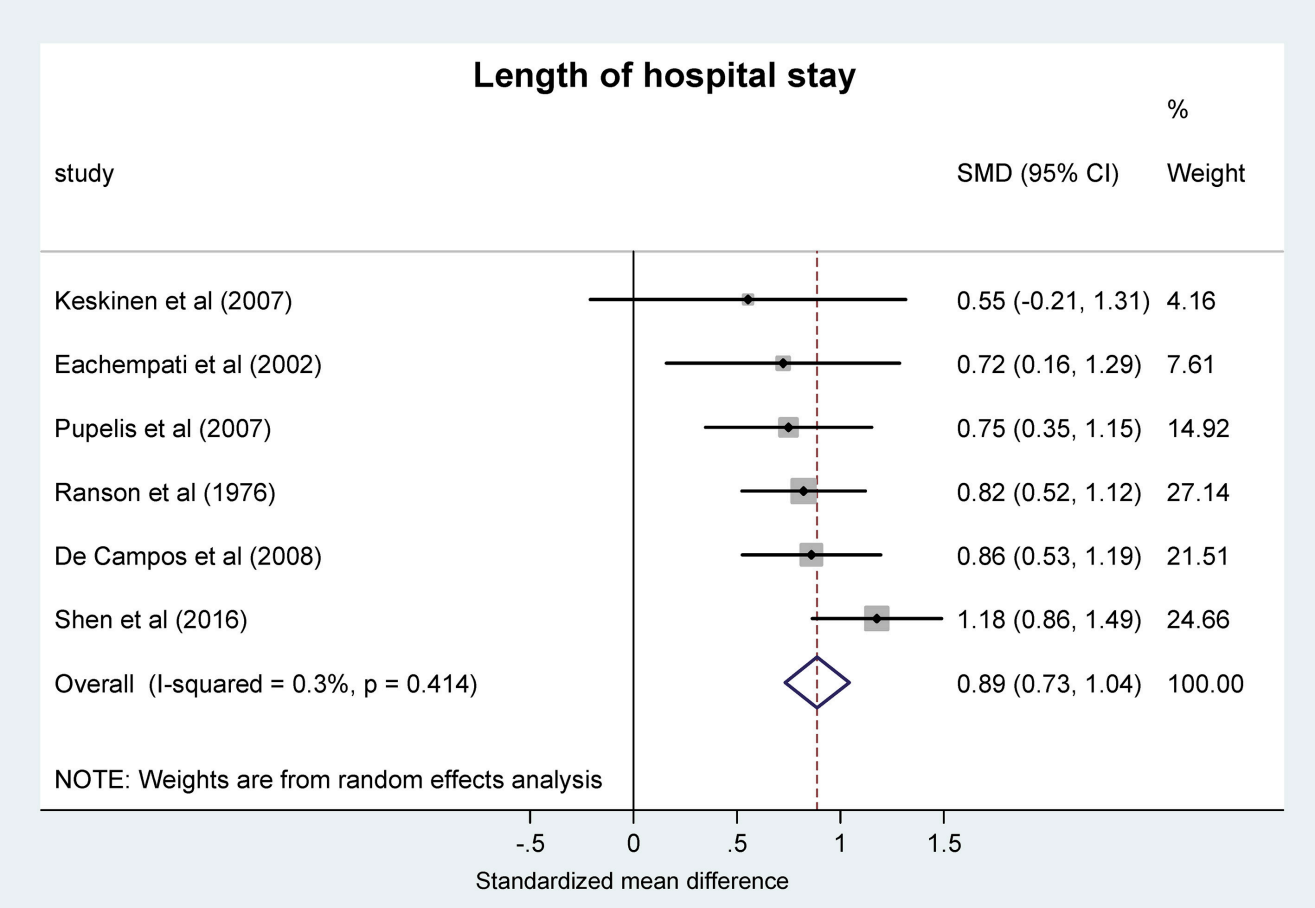
Forest plot analysis of the length of hospital stay using random-effects model in different systemic pH groups of patients with acute pancreatitis. For explanation, see the legend of Figure 3. SMD, standardized mean difference; CI, confidence interval.

Next, we analyzed the LOS in patients with AP by using the same grouping of acid-base status as used for mortality and severity scores. For the meta-analysis, LOS was expressed as SMD between the patient groups. We found that the pooled difference was significantly positive between the more acidotic patient groups and the groups with higher pH or bicarbonate concentrations (0.89, 95% CI, 0.73, 1.04; *P* < 0.001) (Figure 4), which difference corresponds to 15.05 days (95% CI, 10.84, 19.19) longer LOS in the more acidotic AP patient group.

#### Meta-regression analysis

**Figure 5:**
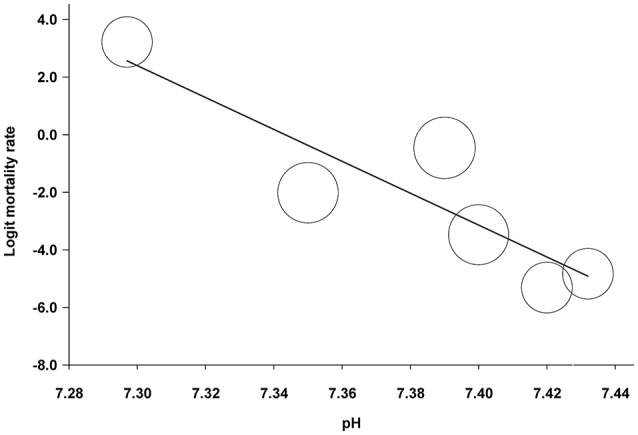
Meta-regression analysis of the association between blood pH and mortality rate in patients with moderately severe and severe forms of acute pancreatitis. The circles indicate estimated logit mortality rate calculated for each patient group. A lower calculated value corresponds with lower mortality rate and vice versa. The circle size is proportional to the precision of the estimated logit mortality rate. The solid black line represents the weighted regression line based on variance-weighted least squares.

As a further statistical approach to determine a correlation between blood pH and mortality in the more progressed forms of AP, we also performed a meta-regression analysis on the collected data. For that, we used those study groups, in which pH and mortality rate was reported in moderately severe or severe manifestations of AP for the same patient groups (Zhu et al., 2003; Lei et al., 2013; Shen et al., 2016). We found a significant correlation between pH and mortality rate with a regression slope of −55.4 (95% CI, −97.9, −12.9; *P* = 0.011) (Figure 5). The potential reason for statistical heterogeneity, as revealed in the forest plots, could not be evaluated in the meta-regression analysis because of the small number of eligible studies.

### Experimental animal model

#### Acidosis is augmented in severe form of AP

**Figure 6:**
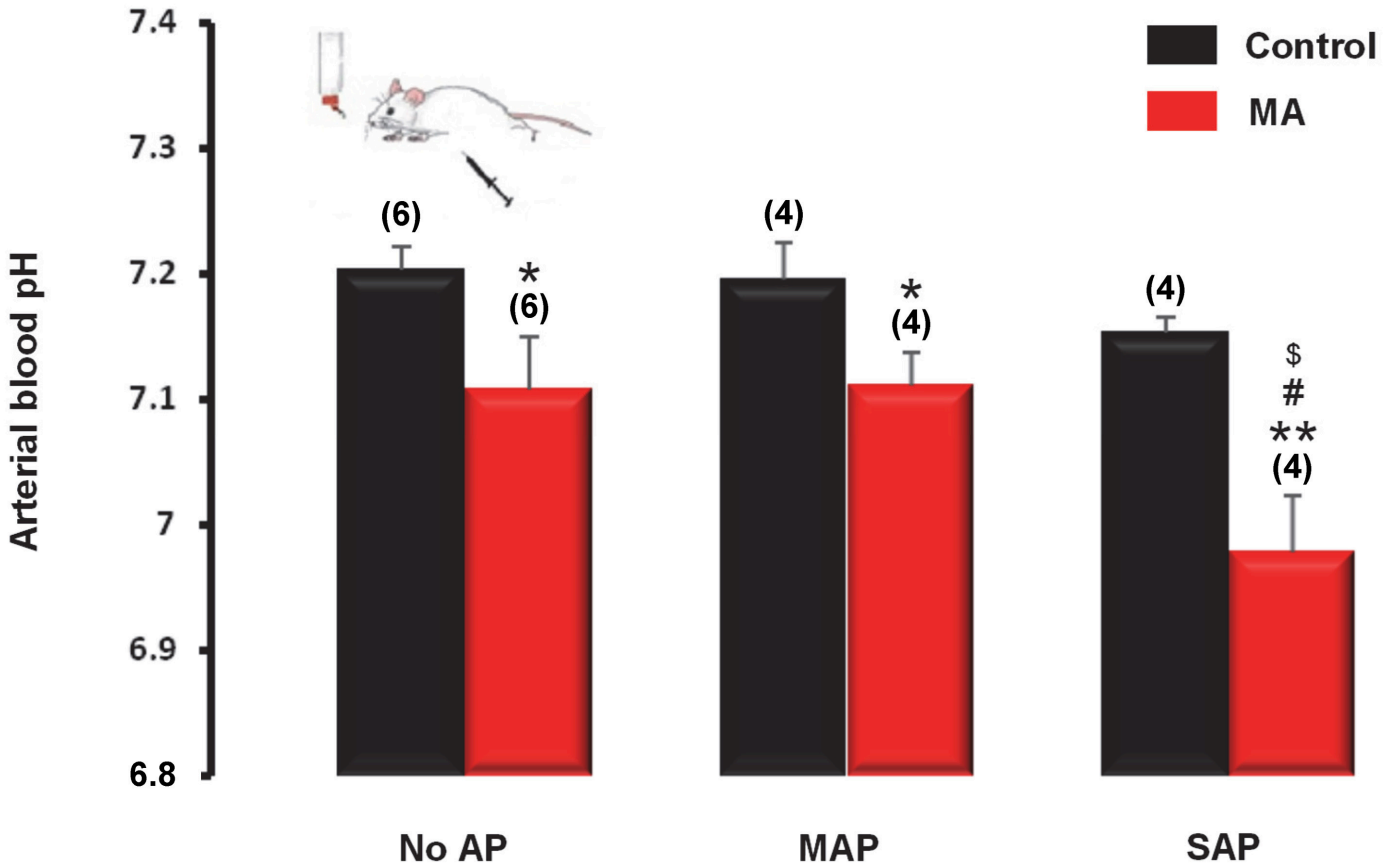
Arterial pH of mice with metabolic acidosis (MA) induced by combination of oral and i.p. NH_4_Cl administration and without acidifying treatment (control). On day 12 of the acidifying treatment, mild acute pancreatitis (MAP) or severe acute pancreatitis (SAP) was induced by alcohol and fatty acid or cerulein, respectively. Mice in the sham pancreatitis group (no AP) were injected i.p. with saline. Statistically significant differences are marked with ^*^between MA and control (non-acidotic) groups, with ^#^between no AP and MAP groups in MA, and with ^$^between no AP and SAP groups in MA, as follows: ^*^P < 0.05 and ^**^P < 0.01 for MA vs. control in no AP, MAP, and SAP; ^#^*P* < 0.05 for no AP in MA vs. SAP in MA; ^$^*P* < 0.05 for MAP in MA vs. SAP in MA.

Analyses of data from 2,311 patients showed strong association between acidosis and the outcome of AP. Therefore, we moved from the “bedside to the bench” to clarify their causative relationship. First, we developed a new experimental model to mimic chronic MA by comparing different types (oral or i.p. or both) of acidifying treatments in mice. We found that MA can be induced in mice by the combined administration of oral and i.p. NH_4_Cl, which decreased blood pH to 6.80 ± 0.04, but it did not cause any pancreatic damage (Supplementary Figure 6), nor did it change serum glucose and urine urea levels (Supplementary Figure 7). Similarly to pH, arterial blood bicarbonate level decreased most significantly (*P* < 0.001) in the combination (oral and i.p.) treatment group as compared to controls (16.5 ± 0.9 vs. 26.4 ± 0.8 mmol/l) (Supplementary Figure 7). We detected no significant differences in the serum concentrations of creatinine, sodium, and potassium among the different treatment groups (data not shown). We used this MA model to study the interaction between acidosis and AP. For that, mice with or without pre-existing MA were assigned to MAP, SAP, and control (no AP) groups and their arterial blood pH were compared. As expected, pre-existing MA induced by dual (oral and i.p.) acidifying treatment resulted in significantly decreased blood pH in the mice without AP, as well as in mice with either MAP or SAP (Figure 6). In the mice with pre-existing MA, the extent of the pH reduction was similar in the sham AP and MAP groups (7.08 ± 0.04 and 7.11 ± 0.03, respectively), while in the mice with SAP the arterial pH decreased to 6.97 ± 0.05, which was significantly lower than in the sham AP and MAP groups (*P* < 0.05 compared to both), suggesting that SAP further deteriorates MA (Figure 6). As expected, MA also resulted in decreased arterial blood bicarbonate levels, which reached the level of significance in the MAP group (16.7 ± 1.4 mmol/l; *P* < 0.01) (Supplementary Figure 8). The levels of urea in the urine were markedly decreased in mice with SAP (3.6 ± 0.2 mmol/l) regardless of their pH status as compared to the urine urea levels in the sham AP groups without and with pre-existing MA (7.4 ± 0.2 and 7.5 ± 0.1 mmol/l, respectively; *P* < 0.001 for both). Importantly, MA significantly lowered urine urea levels in mice with MAP compared to mice with MAP without pre-existing MA (5.9 ± 0.7 vs. 8.9 ± 1.0 mmol/l; *P* < 0.05) (Supplementary Figure 8). We did not detect significant difference in the serum concentrations of glucose (Supplementary Figure 8), and in the levels of creatinine, sodium, and potassium among the different treatment groups (data not shown).

#### Pre-existing acidosis deteriorates both mild and severe forms of AP in mice

**Figure 7:**
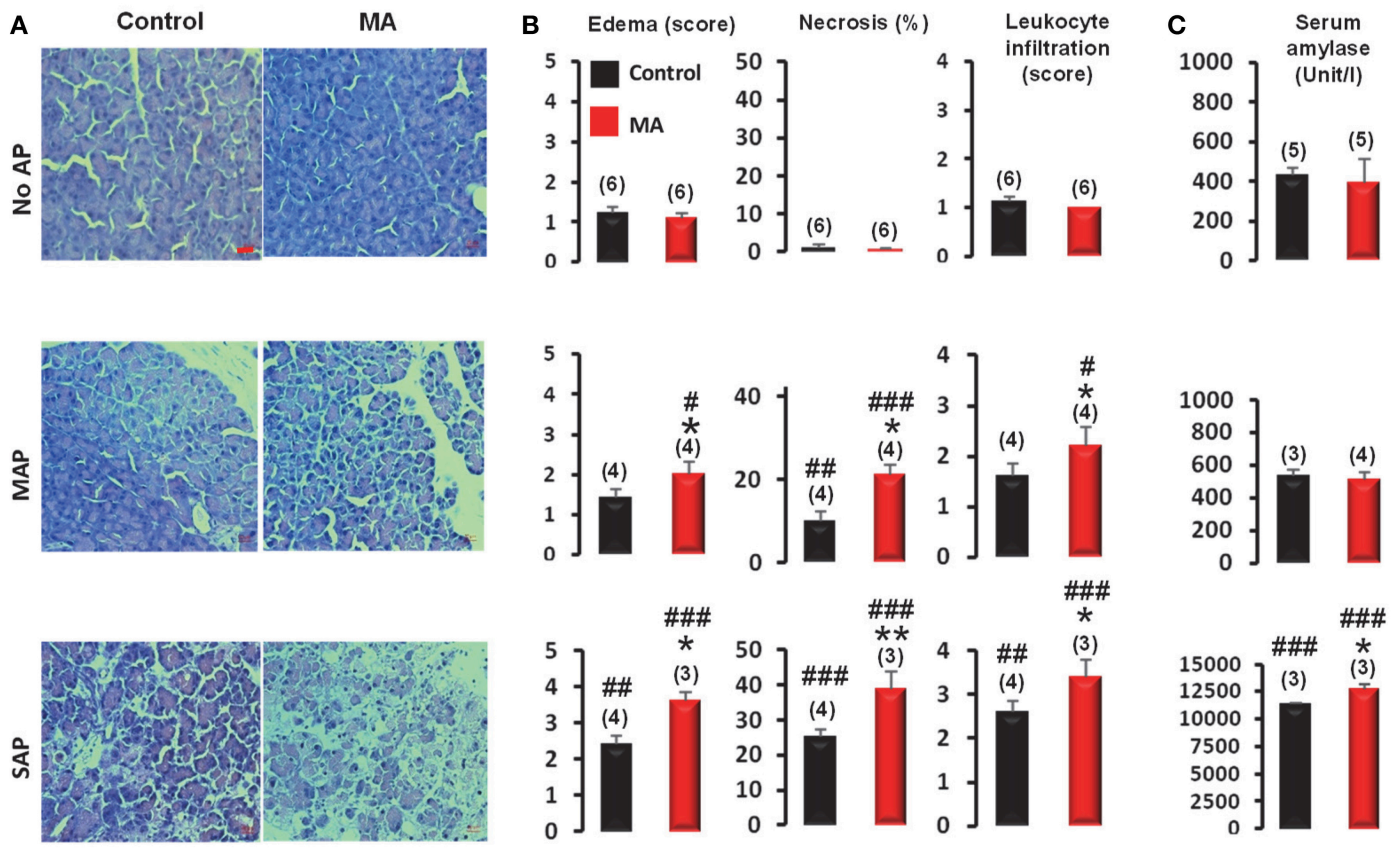
Assessment of the severity of acute pancreatitis (AP) in mice with and without metabolic acidosis (MA and control, respectively). **(A)** Representative microphotographs of pancreatic sections, **(B)** histological evaluation of edema scores, necrosis, and leukocyte infiltration scores, and **(C)** serum amylase levels of MA and control mice with mild acute pancreatitis (MAP), severe acute pancreatitis (SAP) or without acute pancreatitis (no AP). Scale bar represents 20 μm. Statistically significant differences are marked with ^*^between MA and control (non-acidotic) groups and with ^#^between no AP and either MAP or SAP groups, as follows: ^*^P < 0.05; ^**^P < 0.01 for MA vs. control in MAP and SAP; ^#^*P* < 0.05, ^##^*P* < 0.01, and ^###^*P* < 0.001 for no AP vs. MAP and SAP.

To determine whether the presence of pre-existing MA has any effects on the pancreatic damage during AP, pancreatic edema, necrosis, and leukocyte infiltration scores were assessed in pancreatic sections of mice without AP, or with MAP or SAP in the presence and the absence of pre-existing MA. The acidifying treatment caused no pancreatic damage in the control (no AP) mice (Figure 7), which is line with our previous results (Supplementary Figure 6). On the contrary, in mice with pre-existing MA, MAP resulted in significantly larger edema (2.0 ± 0.3 vs. 1.4 ± 0.2; *P* < 0.05), increased necrosis (21.0 ± 2.4 vs. 10.0 ± 2.2%; *P* < 0.05), and elevated leukocyte infiltration (2.5 ± 0.4 vs. 1.6 ± 0.2; *P* < 0.05) compared to MAP in mice with normal blood pH (Figure 7). Pancreatic damage was also markedly more pronounced in SAP in mice with pre-existing MA compared to SAP in mice with normal blood pH as indicated by increased edema (3.6 ± 0.2 vs. 2.4 ± 0.2; *P* < 0.05), necrosis (38.6 ± 5.0 vs. 25 ± 2.2%; *P* < 0.01), leukocyte infiltration (3.6 ± 0.4 vs. 2.6 ± 0.2; *P* < 0.05), and serum amylase activity (12,730 ± 384 vs. 11,362 ± 106 Unit/l; *P* < 0.05) (Figure 7). These results suggest that MA further deteriorates pancreatic damage in both MAP and SAP.

## Discussion

In the present study, we revealed a strong association between blood pH and the outcome of AP with meta-analysis of human studies. Our analyses showed that lower blood pH predicts higher mortality rate, longer LOS, and worsens the severity of AP. A significant negative correlation between blood pH and mortality rate in severe forms of AP was found with meta-regression analysis of the human studies. To better clarify how MA can interact with AP, we developed a mouse model of chronic MA and showed that SAP worsens the MA in the mice. In the same model we also demonstrated that pre-existing MA further deteriorates the tissue damage in both mild and severe forms of AP.

Although previous human studies indicated a link between MA and AP (Nair et al., 2000; Zhu et al., 2003; Shinzeki et al., 2008; Sharma et al., 2014; Shen et al., 2016), we found only one, single-center prospective study which directly aimed to explore this correlation (Sharma et al., 2014). Because of the scarcity of data available from targeted clinical trials, we aimed to clarify the association between MA and AP by systematic review of the current literature and by meta-analysis of the available data. By identifying 13 eligible studies for the analysis (Ranson et al., 1976; Nair et al., 2000; Eachempati et al., 2002; Zhu et al., 2003; Kaya et al., 2007; Keskinen et al., 2007; Pupelis et al., 2007; De Campos et al., 2008; Shinzeki et al., 2008; Lei et al., 2013; Sharma et al., 2014; Zhan et al., 2015; Shen et al., 2016), we included 2,311 patients with AP in the analyses. In all of these studies, blood sample analysis was performed at admission or within 24 h thereafter, hence the blood pH parameters were determined with practically the same latency compared to the time when AP was diagnosed. Unavoidably however, the disease could progress to different stages in the different patients before the diagnosis has been reached. There were huge differences between the protocols of the individual studies, but it is remarkable that no matter how the patients were grouped by the authors originally, the patient group with lower pH had always (with no exceptions) worse outcomes (mortality rate, LOS, severity scores) than the group with higher pH in AP, which suggests that in the early stages (viz., until the time of diagnosis) of AP acidosis is an important influencing factor of the outcome regardless from the actual progression of the disease. Unfortunately, the design of the studies did not allow to analyze the causative relationship between MA and AP. In most of the studies, the systemic pH status of the patients prior to or repeatedly after the diagnosis of AP was not reported, thus the dynamics in the changes of pH during the time course of AP could not be assessed in the current analysis, but it is notable that the average base deficit was markedly (4-8 fold) higher in populations of patients, who did not survive SAP (Kaya et al., 2007; Keskinen et al., 2007). In the prospective trial by Sharma et al. (2014), in those SAP patients, who had a blood pH of less than 7.35, the mortality rate was nearly 10 times higher than in those patients whose pH was above this level (54 vs. 6.5%).

As limitations of our study, it should be mentioned that even though our meta-analysis showed a clear association between blood pH and the outcome of AP, since originally the patients were not divided into subgroups based on their blood pH by the authors, the independent effect of lower blood pH on the outcome and the cause-effect relationship between MA and AP could not be assessed. Because of the same reason and also to reduce the inter-study heterogeneity, in each of the analyzed studies we assigned one patient group as the lower pH group and the other one as the higher pH group. Since the reported pH values differed substantially among the analyzed studies, the cut-off value between the lower and the higher pH groups was individually determined for each study. Consequently, in the present analysis we could not determine a specific cut-off pH value which would be detrimental for the outcome of AP. The most convincing method to obtain direct evidence for the role of acidosis as an independent risk factor in AP, determine a detrimental cut-off pH value, and gain insight into the cause-effect relationship in humans would be to conduct targeted clinical trials in which patients with AP are grouped based on their blood pH at admission and their acid-base status as well as the severity and the outcome of AP is continuously monitored. By collecting data of individual patients in such clinical trials, it would be possible to statistically analyze the direct (independent) effect of acid-base disturbances on AP. Until such or similar trials are conducted, we are restricted to use different (not so direct) approaches such as meta-analyses and animal experiments.

To discover whether a pre-existing MA worsens the outcome of AP or MA is rather the result of the progression of AP, we moved from the “bedside to the bench.” Gorelick's workgroup has discovered that low extracellular pH induces pathophysiological changes in acinar cells (Bhoomagoud et al., 2009; Reed et al., 2011). They described that reduced pH sensitizes the acinar cell to secretagogue-induced pancreatitis responses in rats, and enhances connexin32 degradation and ryanodine receptor-mediated calcium signaling in the basolateral region of the acinar cell which mechanisms are responsible for the injurious effects of low extracellular pH on the exocrine pancreas (Bhoomagoud et al., 2009; Reed et al., 2011, 2014). However, the authors have not investigated the causative relationship between low pH and pancreatitis. Since no mouse model of chronic MA was available in the literature, first we designed a set of experiments to develop the most suitable MA model. Dual administration (oral and i.p.) of acidic fluid induced a marked pH drop in the blood without damaging the pancreas. By supplementing the oral treatment with i.p. acidification, our model also accounted for such conditions, when primarily the pH of the peritoneal fluid is reduced such as bacterial peritonitis (Glinska-Suchocka et al., 2016), carbon dioxide insufflation during laparoscopy (Duerr et al., 2008), or peritoneal dialysis (Farhat et al., 2008). Notably, in our model, MA developed gradually and persisted for several days in the mice which is very similar to the development of MA in human patients. Indeed, there is evidence that in human patients AP can develop in pre-existing MA, for instance in diabetic ketoacidosis either via hyperlipidemia (Nair and Pitchumoni, 1997; Nair et al., 2000) or through distinct mechanisms (Gianfrate and Ferraris, 1998). It should be noted however that in clinical settings MA typically occurs as a consequence of AP and in many cases it does not pre-exist. The shown reverse relationship between MA and AP, namely that the presence of a pre-existing acidosis can influence the severity of AP, warrants for careful pH management in such clinical situations.

Sodium bicarbonate therapy is widely accepted for the treatment of MA in conditions associated with the loss of bicarbonate (e.g., renal tubular acidosis, diarrhea), however its use to increase pH in diseases associated with acidosis not due to bicarbonate loss is questionable because of its adverse effects, for example, intracellular acidosis, hypokalemia, and decreased serum ionized calcium concentration (for reviews, see Adeva-Andany et al., 2014; Hopper, 2017). In AP, bicarbonate production becomes impaired due to the damage of the pancreatic tissue, hence when systemic pH decreases bicarbonate administration can be beneficial to maintain normal pH, thereby to improve the outcome based on our results. It has to be noted that, to our knowledge, currently there is no evidence for the benefits of bicarbonate administration in AP. In contrast with sodium bicarbonate therapy, a growing body of evidence supports the beneficial effects of lactated Ringer's solution in the treatment of AP. Since lactate is metabolized to bicarbonate in the liver, lactated Ringer's solution was successfully used to lessen the metabolic acidosis by elevating blood bicarbonate levels and to attenuate the systemic inflammation response as assessed by lower C-reactive protein (CRP) levels in patients with AP (Wu et al., 2011; de-Madaria et al., 2018). Administration of lactated Ringer's solution resulted in lower mortality rate in critically ill patients with AP (Aboelsoud et al., 2016) and it lead to transiently reduced systemic inflammation in patients with MAP (Choosakul et al., 2018), although it had no therapeutic benefits in AP in a retrospective study (Lipinski et al., 2015). For the initial management of AP, the American College of Gastroenterology guideline recommends lactated Ringer's solution as the preferred isotonic crystalloid fluid replacement with moderate quality of evidence (Tenner et al., 2013). Future clinical trials are warranted to confirm the beneficial effects of tightly controlled pH management and to identify the optimal type of fluid resuscitation in patients with AP and pre-existing MA.

Our experiments clearly showed a strong bilateral link between pH and AP. We showed that pre-existing MA worsens the outcome of AP, whereas AP reduces pH in the blood which vicious cycle could be one of the main reasons for the high mortality rate in AP. The exact mechanism of how MA can deteriorate AP remains subject for future studies, but it can be assumed that complex regulatory mechanisms, such as the pancreatic damage and zymogen activation, neurogenic inflammation, and activation of inflammatory cells and mediators, are involved; for a comprehensive review, see Gorelick and Thrower (2009). Similarly, the question of whether the augmented acidosis is a direct or an indirect consequence (e.g., through impaired kidney and/or lung functions) or a combination of these in AP remains to be answered. Indeed, several complications of AP such as renal, pulmonary, and cardiovascular failure can cause disturbances in the acid-base balance. The development of acute renal dysfunction was reported in several of the analyzed studies (Keskinen et al., 2007; Pupelis et al., 2007; Lei et al., 2013; Sharma et al., 2014; Shen et al., 2016), and the impaired kidney function (determined by increased serum creatinine and blood urea nitrogen levels) was associated with significantly worse outcome, including higher mortality rates in AP (Talamini et al., 1999; Eachempati et al., 2002). Moreover, the frequency of renal failure increased by 5-10 times if acidosis (i.e., blood pH < 7.35, base deficit > 4 mEq/l, or bicarbonate < 22 mEq/l) occurred in AP (Sharma et al., 2014). Since we found only one clinical trial which directly investigated the relationship between renal failure and acidosis in AP (Sharma et al., 2014), the available data in humans were not sufficient for meta-analysis. In our experimental model, acute renal dysfunction occurred in mice with SAP regardless of their pH status, moreover the presence of pre-existing MA significantly impaired the kidney functions in mice with MAP, which is in harmony with the observations in humans and provides direct experimental support to the association between acidosis and renal failure in AP.

Cytokines are important mediators in the whole process of AP. A number of proinflammatory mediators, such as interleukin (IL)-1, 6, 8, and tumor necrosis factor (TNF)-α, were shown to play a role in AP in experimental animals (Norman et al., 1997; Liu et al., 2003; Meng et al., 2005) and in human patients (de Beaux et al., 1996; McKay et al., 1996; Brivet et al., 1999; Mayer et al., 2000). Cytokine production occurs in the pancreas first, and then with the progression of the disease in distant organs like the lungs, liver, and spleen (Norman et al., 1997). The levels of IL-6, 8, and TNF-α are even more increased in SAP than in MAP (McKay et al., 1996; Pooran et al., 2003). Active digestive enzymes which are released from injured pancreatic cells can potently stimulate proinflammatory cytokine production in macrophages (Desser et al., 1994; Lundberg et al., 2000), moreover the pancreatic acinar cells can also produce proinflammatory cytokines (Gukovskaya et al., 1997; Brady et al., 2002). For example, amylase can induce the production of IL-1, 6, and TNF-α in human peripheral blood mononuclear cells and in dermal fibroblasts (Desser et al., 1994; Malpass et al., 2013). Lipase markedly induced TNF-α production in rat macrophages (Jaffray et al., 2000), while CRP was shown to strongly correlate with IL-6 levels in patients with AP (Viedma et al., 1992). In our mouse model, we found that amylase level was elevated in SAP, and it was further increased in the presence of a pre-existing acidosis, therefore it can be expected that circulating cytokine levels are also higher in the co-existence of SAP and MA than in SAP without acidosis. In patients with SAP, the serum lipase and CRP levels were higher when their blood pH was lower (Pupelis et al., 2007), thus suggesting higher levels of circulating cytokines.

The production of pro- and anti-inflammatory cytokines was repeatedly shown to depend from the extracellular pH (for reviews, see Kellum et al., 2004b; Okajima, 2013; Casimir et al., 2018). Although the different forms and severities of acidosis can differently influence cytokine production (Kellum et al., 2004b), a proinflammatory effect, including enhanced TNF-α synthesis and augmented nuclear factor-κB activation, of hyperchloremic acidification was shown in activated macrophages by independent groups (Bellocq et al., 1998; Heming et al., 2001; Kellum et al., 2004a). Also, decreasing extracellular pH caused increasing IL-8 expression and nuclear factor-κB activation in human pancreatic tumor cells (Shi et al., 2000). In addition to the recruitment of immune cells by the low pH-induced cytokine release, extracellular acidosis *per se* promotes the activation of neutrophils (Martinez et al., 2006), which is in line with the increased leukocyte infiltration in MAP and SAP with pre-existing acidosis compared to MAP and SAP with initially normal blood pH, as observed in the present study (Figure 7B). Here, we revealed a bidirectional relationship constituting a vicious circle between AP and acidosis and developed a mouse model for studying the underlying mechanisms of the progression of AP in pre-existing MA. However, the experimental conformation of the dynamics of tissue and circulating cytokine concentrations and other potential processes (e.g., calcium signaling) in this model remains subject for future studies.

In summary, by the meta-analysis of literature data available from human studies we found a significant correlation between low systemic pH and the outcome of AP, indicating that lower pH level is associated with higher mortality rates, longer LOS, and more severe AP. With regards to the mechanism, in experimental animals we showed the existence of a bidirectional interaction between MA and AP, in which pre-existing MA deteriorates AP and, vice versa, AP further increases the severity of MA. Our findings suggest that systemic pH level should be closely monitored in patients with AP and that interventions to normalize the low pH of patients with AP should be considered in clinical settings. Well-designed, targeted clinical trials are warranted to evaluate the effects of the therapeutic interventions of acidosis in patients with AP.

## Author contributions

ZRu and AG conducted the literature search of meta-analysis and the quality assessment of included studies, and extracted data from the articles. ET, JM, ZB, and PH performed the experiments. PH and AG conceived and supervised the meta-analysis and the experimental procedures and obtained funding. ZRu, ET, LP, JM, AG, and PH analyzed and interpreted the data. ZRu, ET, LP, PH, and AG wrote the paper. LP, EO, AV, GV, LC, KM, AM, ZRa, ZB, JK, IF, and JM reviewed and contributed to the manuscript. All authors approved the final manuscript.

### Conflict of interest statement

The authors declare that the research was conducted in the absence of any commercial or financial relationships that could be construed as a potential conflict of interest.

## Abbreviations

AP: acute pancreatitis
APACHE: acute physiology and chronic health evaluation
CI: confidence interval
CRP: C-reactive protein
ES: estimated logit mortality rate
IL: interleukin
i.p.: intraperitoneal(ly)
LOS: length of hospital stay
MA: metabolic acidosis
MAP and SAP, mild and severe acute pancreatitis: respectively
SEM: standard error of mean
SMD: standardized mean difference
TNF: tumor necrosis factor.
