# Supplementary material for: Bidirectional Relationship Between Reduced Blood pH and Acute Pancreatitis: A Translational Study of Their Noxious Combination
Source: Front Physiol. 2018 Oct 1;9:1360. doi: 10.3389/fphys.2018.01360 (PMC6174522; doi:10.3389/fphys.2018.01360)
Supplement: Supplementary file 1 [file Image_1.pdf]

Supplementary material

Supplementary Table 1. PRISMA checklist.

Section/topic	#	Checklist item	Reported on page #
**TITLE**			
Title	1	Identify the report as a systematic review, meta-analysis, or both.	not applicable
**ABSTRACT**			
Structured summary	2	Provide a structured summary including, as applicable: background; objectives; data sources; study eligibility criteria, participants, and interventions; study appraisal and synthesis methods; results; limitations; conclusions and implications of key findings; systematic review registration number.	1
**INTRODUCTION**			
Rationale	3	Describe the rationale for the review in the context of what is already known.	2
Objectives	4	Provide an explicit statement of questions being addressed with reference to participants, interventions, comparisons, outcomes, and study design (PICOS).	2
**METHODS**			
Protocol and registration	5	Indicate if a review protocol exists, if and where it can be accessed (e.g., Web address), and, if available, provide registration information including registration number.	2
Eligibility criteria	6	Specify study characteristics (e.g., PICOS, length of follow-up) and report characteristics (e.g., years considered, language, publication status) used as criteria for eligibility, giving rationale.	2
Information sources	7	Describe all information sources (e.g., databases with dates of coverage, contact with study authors to identify additional studies) in the search and date last searched.	2
Search	8	Present full electronic search strategy for at least one database, including any limits used, such that it could be repeated.	2
Study selection	9	State the process for selecting studies (i.e., screening, eligibility, included in systematic review, and, if applicable, included in the meta-analysis).	2
Data collection process	10	Describe method of data extraction from reports (e.g., piloted forms, independently, in duplicate) and any processes for obtaining and confirming data from investigators.	2
Data items	11	List and define all variables for which data were sought (e.g., PICOS, funding sources) and any assumptions and simplifications made.	2-3
Risk of bias in individual studies	12	Describe methods used for assessing risk of bias of individual studies (including specification of whether this was done at the study or outcome level), and how this information is to be used in any data synthesis.	3

Summary measures	13	State the principal summary measures (e.g., risk ratio, difference in means).	3
Synthesis of results	14	Describe the methods of handling data and combining results of studies, if done, including measures of consistency (e.g., I^2) for each meta-analysis.	3
Risk of bias across studies	15	Specify any assessment of risk of bias that may affect the cumulative evidence (e.g., publication bias, selective reporting within studies).	3
Additional analyses	16	Describe methods of additional analyses (e.g., sensitivity or subgroup analyses, meta-regression), if done, indicating which were pre-specified.	3
**RESULTS**			
Study selection	17	Give numbers of studies screened, assessed for eligibility, and included in the review, with reasons for exclusions at each stage, ideally with a flow diagram.	4; Figure 1
Study characteristics	18	For each study, present characteristics for which data were extracted (e.g., study size, PICOS, follow-up period) and provide the citations.	4; Figure 1; Supplementary table 3
Risk of bias within studies	19	Present data on risk of bias of each study and, if available, any outcome level assessment (see item 12).	3; Supplementary table 2
Results of individual studies	20	For all outcomes considered (benefits or harms), present, for each study: (a) simple summary data for each intervention group (b) effect estimates and confidence intervals, ideally with a forest plot.	4; Figures 2-4
Synthesis of results	21	Present results of each meta-analysis done, including confidence intervals and measures of consistency.	4
Risk of bias across studies	22	Present results of any assessment of risk of bias across studies (see Item 15).	4; Supplementary figures 1-5
Additional analysis	23	Give results of additional analyses, if done (e.g., sensitivity or subgroup analyses, meta-regression [see Item 16]).	4; Figure 5
**DISCUSSION**			
Summary of evidence	24	Summarize the main findings including the strength of evidence for each main outcome; consider their relevance to key groups (e.g., healthcare providers, users, and policy makers).	6
Limitations	25	Discuss limitations at study and outcome level (e.g., risk of bias), and at review-level (e.g., incomplete retrieval of identified research, reporting bias).	8-10
Conclusions	26	Provide a general interpretation of the results in the context of other evidence, and implications for future research.	11
**FUNDING**			
Funding	27	Describe sources of funding for the systematic review and other support (e.g., supply of data); role of funders for the systematic review.	11

Supplementary Table 2. Quality assessment of the studies included in the meta-analysis with the Newcastle-Ottawa Scale. A score of 7 to 9 indicates high methodological quality, a score of 4 to 6 indicates moderate quality and score of 0 to 3 indicates low quality.

First author	Year	Selection	Comparability	Outcome/Exposure	Total score
**De Campos**	**2008**	***	**	***	**8**
**Eachempati**	**2002**	***	**	***	**8**
**Kaya**	**2007**	***	**	***	**8**
**Keskinen**	**2007**	**	**	*	**5**
**Lei**	**2013**	****	**	***	**9**
**Nair**	**2000**	***	**	***	**8**
**Pupelis**	**2007**	***	**	***	**8**
**Ranson**	**1976**	***	**	***	**8**
**Sharma**	**2014**	***	*	**	**6**
**Shen**	**2016**	***	**	***	**8**
**Shinzeki**	**2008**	**	**	**	**6**
**Zahn**	**2015**	***	**	***	**8**
**Zhu**	**2003**	**	**	*	**5**

Supplementary Table 3. Summary of study characteristics for publications included in the meta-analyses.

First author	Year	Design	Country	Study period	Sample size (lower pH/ higher pH)	Newcastle-Ottawa score
Ranson	1976	Retro- and prospective	USA	01/1971-02/1975	300 (56/244)	8
Nair	2000	Prospective observational	USA	01/1998-02/1999	90 (11/-)	8
Eachempati	2002	Prospective	USA	01/1993-05/2001	76 (16/60)	8
Zhu	2003	Retrospective	China	01/1993-12/2002	74 (12/62)	5
Kaya	2007	Prospective	Turkey	1998-2002	199 (27/172)	8
Keskinen	2007	Retrospective	Finland	2001-2003	59 (9/28)	5
Pupelis	2007	Prospective	Latvia	2000-2005	111 (72/39)	8
De Campos	2008	Retro- and prospective	Brazil	01/1999-11/2005	71 (47/24)	8
Shinzeki	2008	Prospective	Japan	07/1995-06/2006	93 (5/88)	6
Lei	2012	Retrospective	China	04/2007-07/2010	184 (51/133)	9
Sharma	2014	Prospective single center	India	01/2012-11/2013	205 (35/170)	6
Zhan	2015	Retrospective	China	07/2006-06/2010	101 (18/83)	8
Shen	2016	Retrospective cohort	China	11/2010-06/2014	186 (85/101)	8

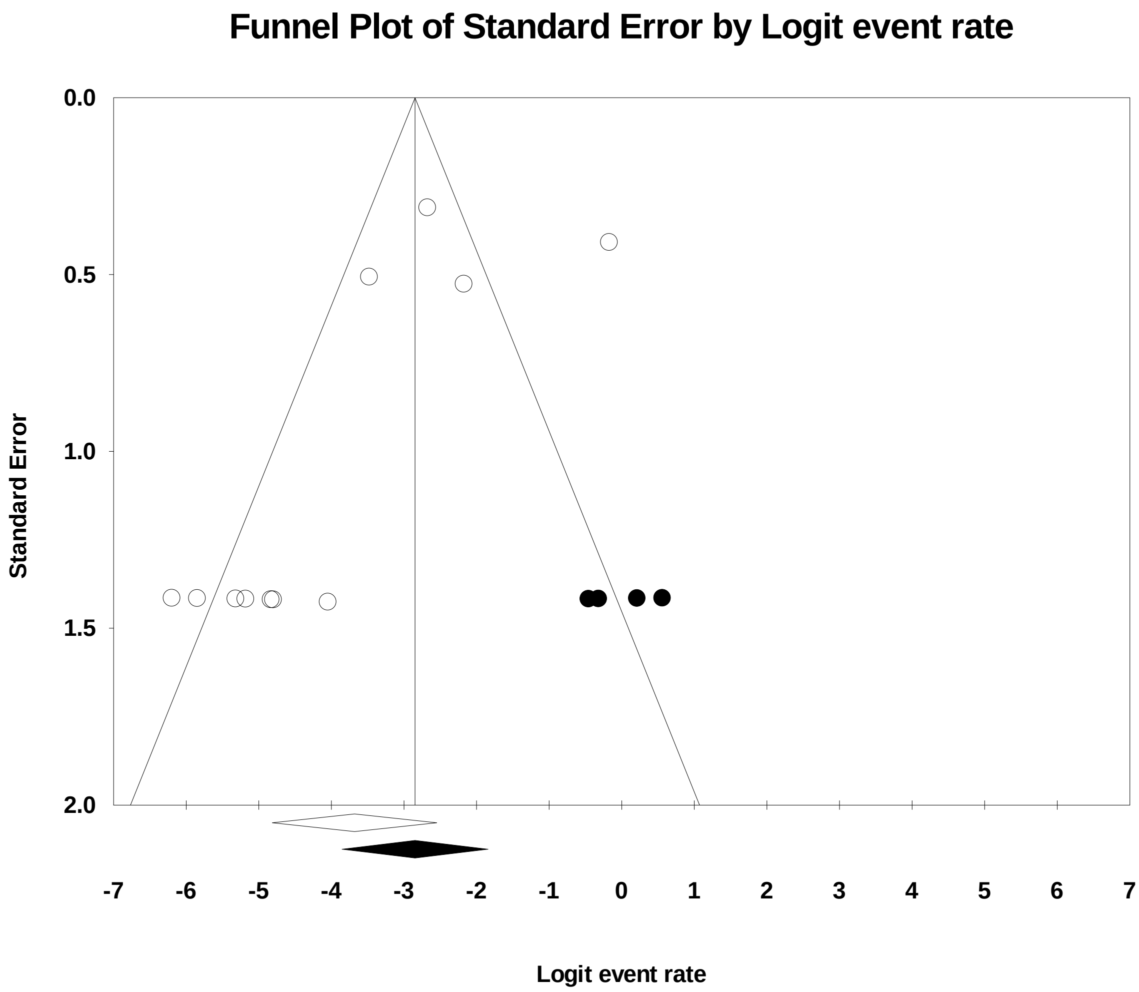

Supplementary Figure 1. Funnel plot of the studies that were included in the forest plot of mortality rate and reported higher systemic pH in patient groups with acute pancreatitis (AP). Here and in Supplementary Figures 2-5, open symbols represent results from studies included in the forest plot, while closed symbols indicate studies that appeared to be missing according to the trim and fill method of Duval and Tweedie. Open and closed diamonds represent the average estimated effect size without (open diamond) and with trim and fill correction (closed diamond). Duval and Tweedie corrected value: -2.85 (95% CI, -3.85, -1.84); *P* = 0.029 with Egger’s test.

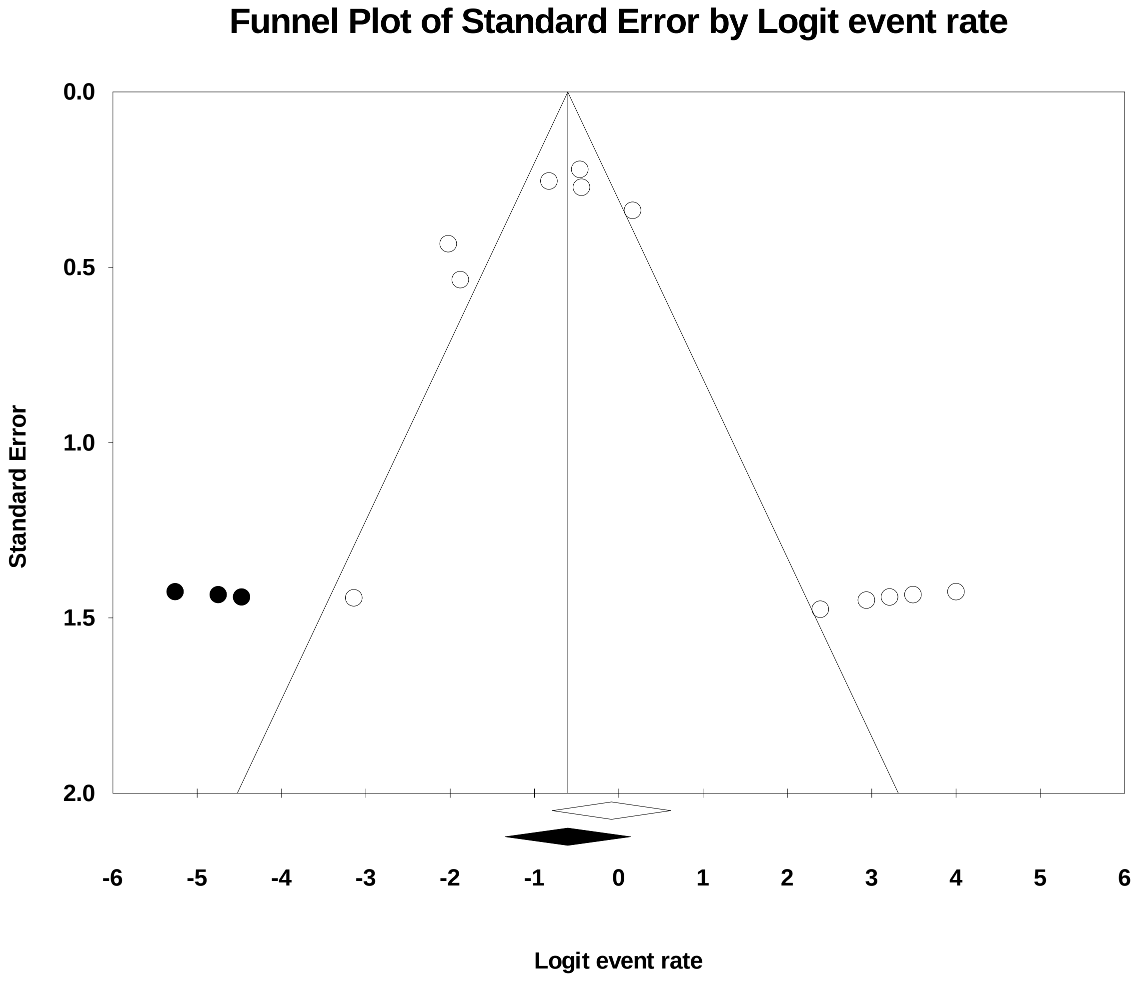

Supplementary Figure 2. Funnel plot of the studies that were included in the forest plot of mortality rate and reported lower systemic pH in patient groups with AP. Duval and Tweedie trim and fill corrected value: -0.615 (95% CI, -1.36, 0.134); *P* = 0.154 with Egger's test.

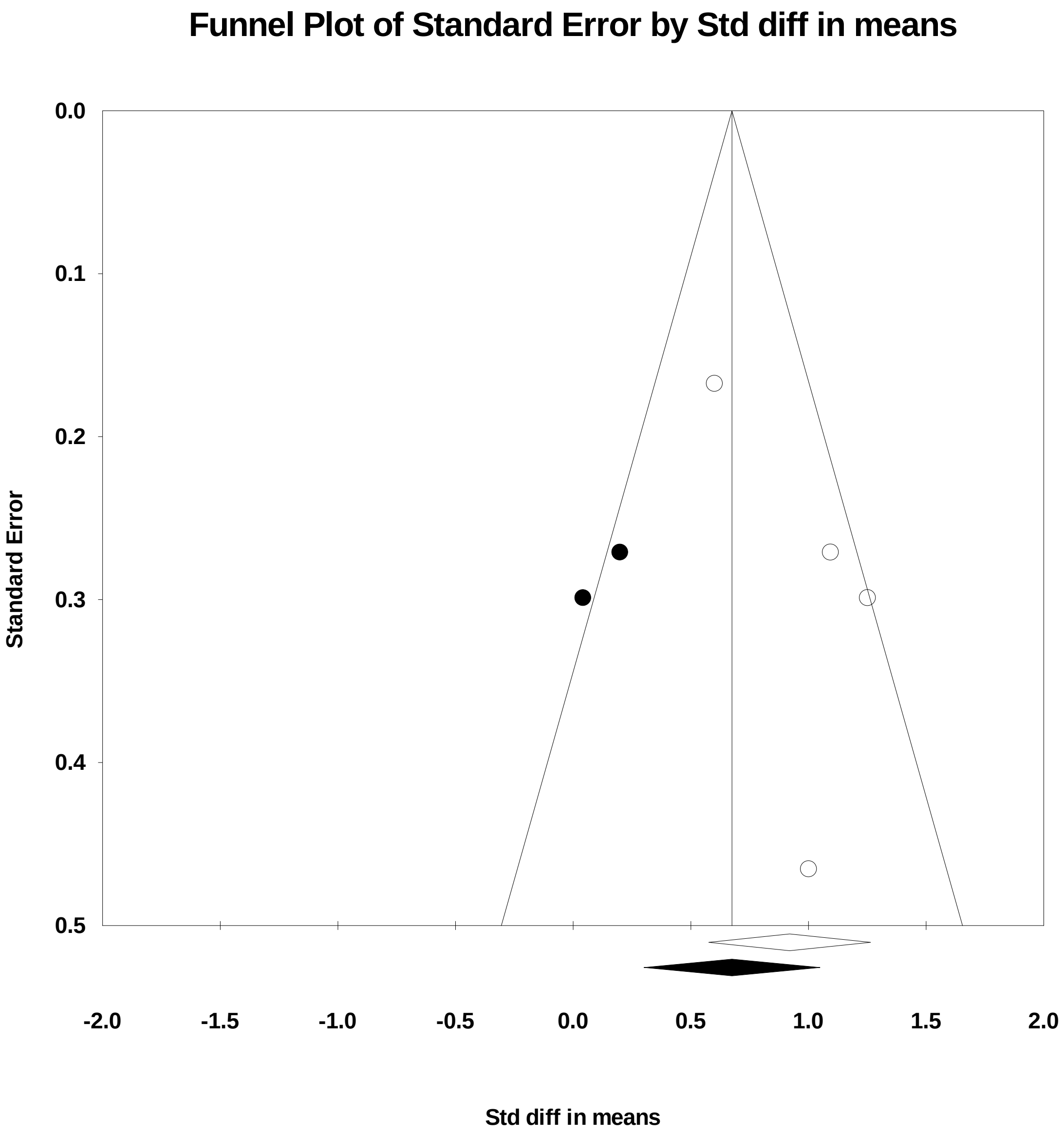

Supplementary Figure 3. Funnel plot of the studies that were included in the forest plot of the Ranson score in different systemic pH groups of patients with AP. Duval and Tweedie trim and fill corrected value: 0.675 (95% CI, 0.30, 2.06); P = 0.234 with Egger's test.

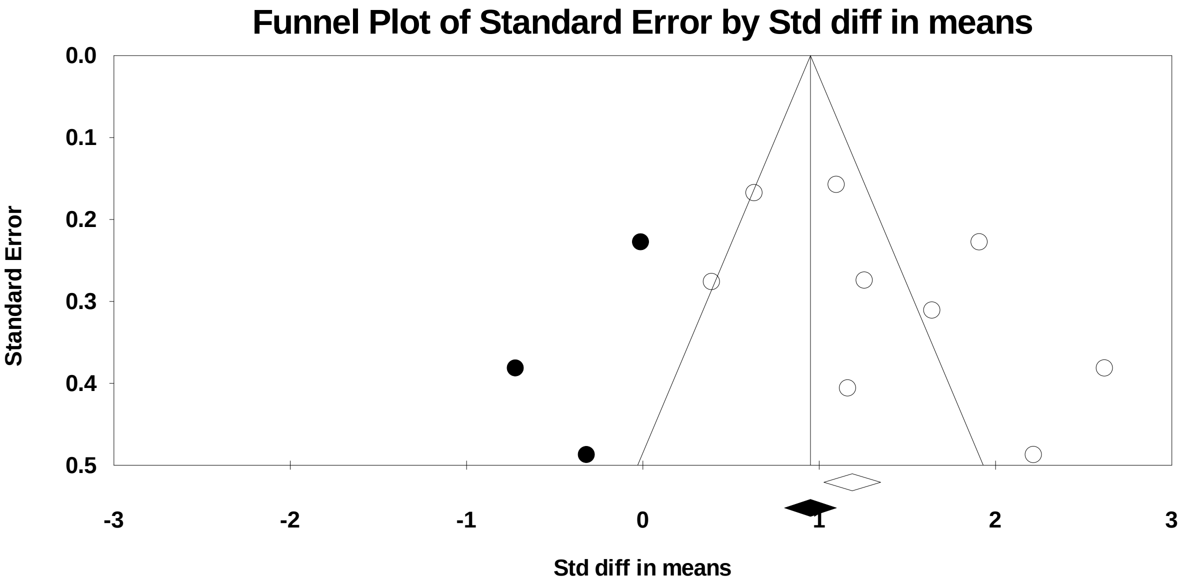

Supplementary Figure 4. Funnel plot of the studies that were included in the forest plot of the Acute Physiology and Chronic Health Evaluation (APACHE II) score in different systemic pH groups of patients with AP. Duval and Tweedie trim and fill corrected value: 0.99 (95% CI, 0.52, 1.46); *P* = 0.138 with Egger's test.

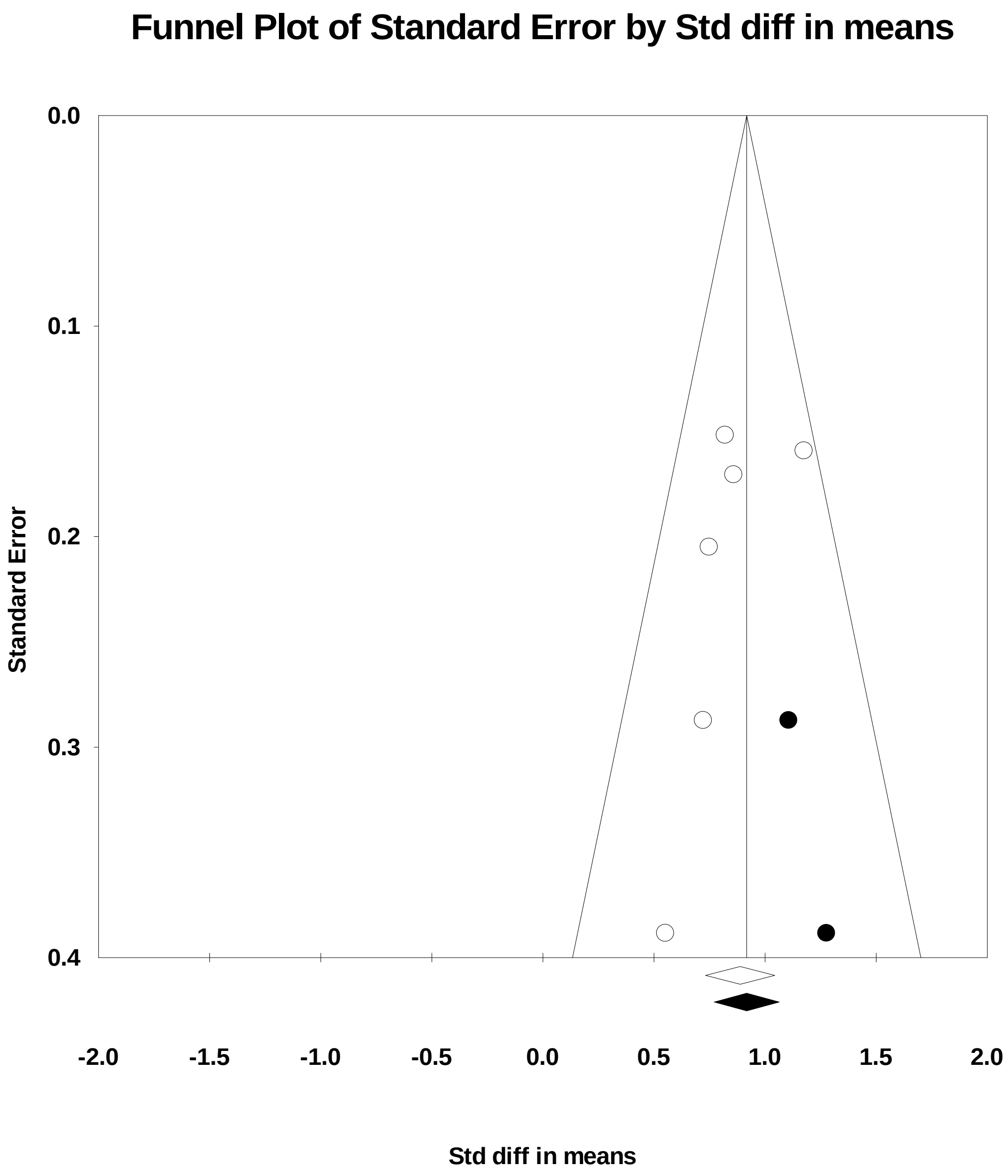

Supplementary Figure 5. Funnel plot of the studies that were included in the forest plot of the length of hospital stay (LOS) in different systemic pH groups of patients with AP. Duval and Tweedie trim and fill corrected value: 0.917 (95% CI, 0.771, 1.064); *P* = 0.231 with Egger's test.

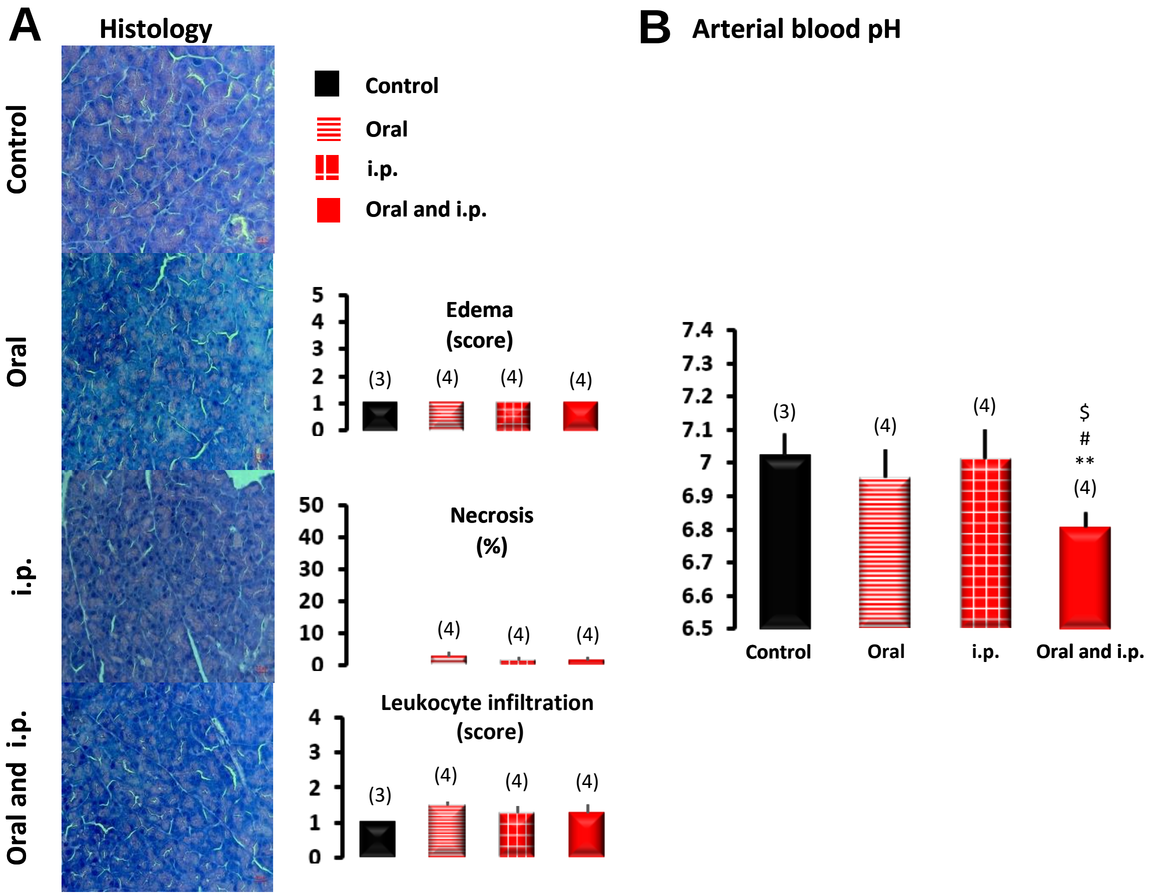

Supplementary Figure 6. Induction of metabolic acidosis with different types (oral, i.p. or both) of NH_4Cl administration in mice. (**A**) None of the treatments caused pancreatic edema or necrosis, and only minimal (not significant) increase of leukocyte infiltration was observed in all three NH_4Cl treatment groups compared to the controls. (**B**) Arterial blood pH decreased minimally in the oral or i.p. treatment groups, while the combined (oral and i.p.) treatment significantly decreased arterial pH. **, $P < 0.01$ for the control group versus the oral and i.p. group; $^{\#}$, $P < 0.05$ for the oral group versus the oral and i.p. group; and $^{\$}$, $P < 0.05$ for the i.p. group versus the oral and i.p. group. Scale bar represents 20 µm. Here and in Supplementary Figures 7 and 8, numbers in parentheses indicate the number of animals in the corresponding groups.

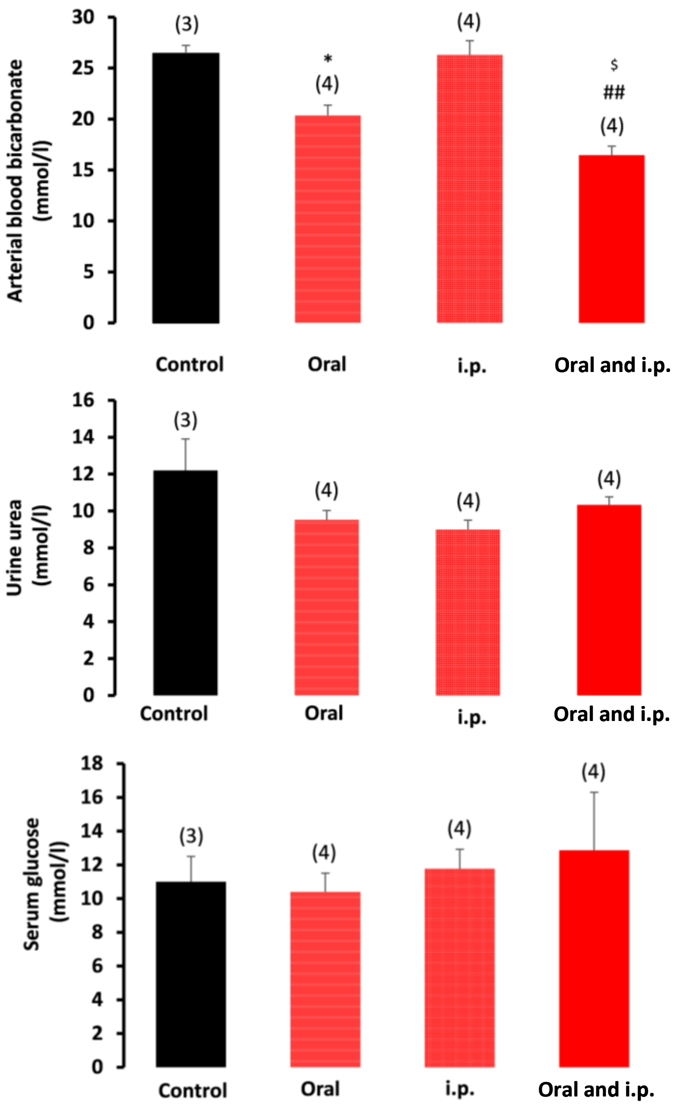

Supplementary Figure 7. Arterial blood bicarbonate, urine urea, and serum glucose levels after different types (oral, i.p. or both) of NH_4Cl administration in mice. Arterial blood bicarbonate level decreased minimally in the i.p. treatment group, while the oral and the combined (oral and i.p.) treatment significantly decreased arterial bicarbonate level. Serum glucose and urine urea levels were not changed significantly in the treatment groups. *, $P < 0.05$ for the control group versus the oral group; $^{\#\#}$, $P < 0.001$ for the control group versus the oral and i.p. group; and $^{\$}$, $P < 0.05$ for the oral group versus the oral and i.p. group.

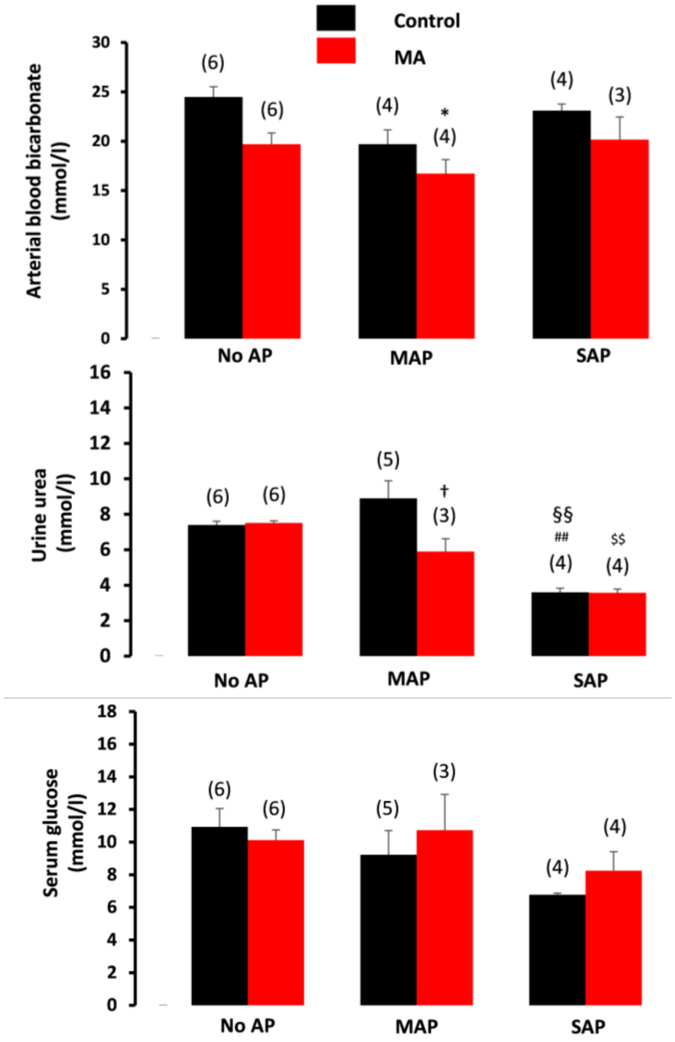

Supplementary Figure 8. Arterial blood bicarbonate, urine urea, and serum glucose levels of mice with and without metabolic acidosis (MA) in sham acute pancreatitis (No AP) and after induction of mild acute pancreatitis (MAP) or severe acute pancreatitis (SAP). Arterial blood bicarbonate and urine urea levels decreased significantly in the MAP and MA group compared to controls. Urine urea levels also decreased in SAP regardless of the pH status. Serum glucose level was not changed significantly. *, $P < 0.05$ for the No AP and control group versus the MAP and MA group; $^{##}$, $P < 0.001$ for the No AP and control group versus the SAP and control group; $^{\$\$}$, $P < 0.001$ for the No AP and MA group versus the SAP and MA group; §§, $P < 0.001$ for the MAP and control group versus the SAP and control group, and †, $P < 0.05$ for the MAP and control group versus the MAP and MA group.
